# Partial Pancreaticoduodenectomy (Whipple Procedure) for Pancreatic Malignancy: Occlusion of a Non-Anastomosed Pancreatic Stump With Fibrin Sealant

**DOI:** 10.1155/1992/48946

**Published:** 1992

**Authors:** Arthur P. Marczell, Michael Stierer

**Affiliations:** Department of Surgery Hanusch Medical Center Heinrich Collin-Strasse 30 Vienna A-1140 Austria

## Abstract

Following partial pancreaticoduodenectomy for periampullary and pancreatic cancer, the complication and mortality rates are particularly high. Various approaches have aimed at improving the postoperative result, with less than complete success. The discouraging results of others, and our own dissatisfaction, led us to evaluate an atraumatic, sutureless method for management of the residual gland. Following head resection, the remaining pancreas is occluded with a fibrin sealant (Tisseel c, Immuno AG, Vienna) via injection into the pancreatic duct, which is then ligated and left free in the peritoneal cavity. Among 44 patients treated with this method, there were no perioperative deaths. Three patients developed local complications (2 fistulae, 1 pancreatitis) due to technical errors that presumably resulted in incomplete occlusion. Evaluation of patients after two to three years indicates that the endocrine function of the pancreas has been largely conserved despite ductal occlusion.


HPB Surgery, 1992, Vol. 5, pp. 251–260
Reprints available directly from the publisher
Photocopying permitted by license only

© 1992 Harwood Academic Publishers GmbH
Printed in the United Kingdom

### PARTIAL PANCREATICODUODENECTOMY (WHIPPLE PROCEDURE) FOR PANCREATIC MALIGNANCY: OCCLUSION OF A NON-ANASTOMOSED PANCREATIC STUMP WITH FIBRIN SEALANT

ARTHUR P. MARCZELL and MICHAEL STIERER
*Department of Surgery, Hanusch Medical Center, Heinrich Collin-Strasse 30, A-1140 Vienna, Austria*

*(Received 1 December 1991)*

Following partial pancreaticoduodenectomy for periampullary and pancreatic cancer, the complication and mortality rates are particularly high. Various approaches have aimed at improving the postoperative result, with less than complete success. The discouraging results of others, and our own dissatisfaction, led us to evaluate an atraumatic, sutureless method for management of the residual gland. Following head resection, the remaining pancreas is occluded with a fibrin sealant (Tisseel c, Immuno AG, Vienna) via injection into the pancreatic duct, which is then ligated and left free in the peritoneal cavity. Among 44 patients treated with this method, there were no perioperative deaths. Three patients developed local complications (2 fistulae, 1 pancreatitis) due to technical errors that presumably resulted in incomplete occlusion. Evaluation of patients after two to three years indicates that the endocrine function of the pancreas has been largely conserved despite ductal occlusion.

KEY WORDS: Complications in pancreatic surgery, Whipple operation, occlusion with fibrin sealant, low complication and mortality rates

### INTRODUCTION

Pancreatic surgery is generally accompanied by a high complication rate and the complication and mortality rates following partial pancreaticoduodenectomy for malignant indication are particularly high[1–9] (Table 1). The average perioperative mortality rate cited for the Whipple procedure has been around 14%, with a range between 3% and 24%, and postoperative complications have been observed in almost half the patients. However, in the past decade morbidity and mortality rates have been falling[3,5,6,8,10].

The poor results seen following the Whipple operation are due largely to leakage from the pancreaticoenteric anastomosis[3,5,6,8,9,10].

A variety of surgical techniques have been tried in order to reduce the incidence of pancreatic fistulas resulting from the lytic effects of pancreatic enzymes on the pancreaticojejunostomy[4,6,8,9,10].

The open pancreatic stump procedure,[11] produced relatively poor results. Its advantage was the absence of perioperative mortality. Splinting (stenting) of the

Address correspondence to: A.P. Marczell MD, Department of Surgery, Hanusch Medical Center, Heinrich Collin-Str. 30, A1140 Vienna, Austria pancreatic duct has also been attempted as a means of protecting the pancreaticoenteric anastomosis from the pancreatic enzymes[6,10].

Pancreatic duct ligation, combined with sealing of the surface of the resection area with fibrin sealant also did not produce the desired result and in some patients, reflux resulted in "retention pancreatitis" and subsequent pancreatic fistulae[12,13].

Some major centers have abandoned the Whipple procedure in favor of total pancreatectomy because of problems related to pancreaticojejunal dehiscence and the possibility of residual cancer in the stump[4,14]. In contrast to van Heerden,[5] we have not encountered evidence of malignant spreads to the pancreatic tail, either on pathologic sections or from clinical evidence. Kummerle was also unable to find evidence of multicentricity[4].

Pharmacologic agents such as somatostatin, terbutaline, glucagon, atropine and calcitonin have been employed to halt pancreatic exocrine activity[15,16]. A new approach, the pancreatic duct occlusion (PDO) with synthetic materials such as prolamine (Ethibloc) and neoprene has been used in both pancreatic cancer and transplantation surgery with reported success in blocking the pancreatic ductal system, but with evidence of occasional transient pancreatic fistulae and/or progressive failure of pancreatic endocrine function[17,18].

A desire to decrease the morbidity and mortality due to leakage at the pancreaticojejunal anastomosis led us to evaluate an atraumatic method for pancreatic stump management which did not involve attachment of the pancreas to the gastrointestinal tract. Based on the knowledge that pancreatic duct stasis leads to atrophy of the exocrine glands, we decided to induce occlusion of the pancreatic ductal system. We chose a physiologic agent that we hoped would not elicit foreign body reaction in the pancreatic stump and subsequent fibrosis of the remaining gland with damage to the islets.

### MATERIAL AND METHOD

### *Fibrin Sealant*

The fibrin sealant (Tisseel c, Immuno AG, Vienna, Austria) has been available for

**Table 1** Perioperative complication and mortality rates associated with pancreaticoduodentectomy for malignant indication

| *Author (Ref.)* | *Year* | *Complications* % | *Mortality* % |
|---|---|---|---|
| Schapiro (1) | 1975 | 50 | 8 |
| Nakase (2) | 1977 | 49 | 21 |
| Warren (3) | 1983 (1951–60) | — | 14 |
| | (1971–80) | — | 3, 4 |
| Kummerle & Ruckert (4) | 1984 | — | 20 |
| Van Heerden (5) | 1984 | 33 | 4 |
| Grace *et al.* (6) | 1986 (1975–79) | 49 | 10 |
| | (1980–84) | 26 | 2 |
| Tarazi *et al.* (7) | 1986 | >40 | 7 |
| Christ *et al.* (8) | 1987 (1969–80) | 59 | 24 |
| | (1981–86) | 36 | 2 |
| Trede & Schwall (9) | 1988 | 32 | 3 |

use in Europe since 1976, in Canada since 1986, and in the United States, under an FDA-approved IND since 1986. It is provided as a multicomponent system from which two solutions are prepared. The sealant is obtained from human plasma and its major constituents are concentrated fibrinogen, factor XIII and fibronectin. Shortly before use, the sealer protein concentrate is dissolved in a solution of an antifibrinolytic agent, aprotinin, which has been warmed to 37<o> C. The clotting activator is dried bovine thrombin dissolved in a calcium chloride solution. A high thrombin concentration (500U/ml) is selected to ensure rapid solidification of the sealant, and a high aprotinin concentration (10<ts>000 U/ml) is used in order to prevent premature dissolution of the fibrin sealant by pancreatic enzymes.

When prepared for use, the solutions are drawn into separate syringes which are then mounted on a special Y-connector which mixes them as they are expelled into the appropriate area of the operating field.

With regard to safety from virus infection, data from the multicenter cardiac study in the United States (Rousou, J., Levitsky, S., Gonzalez-Lavin, L., Cosgrove, D., Magilligan, D., *et al.* Randomized Clinical Trial of Fibrin Sealant in Patients Undergoing Resternotomy of Reoperation after Cardiac Operations. *J. Thorac Cardiovasc. Surg.*, 1989: 97: 194–203) indicated no HIV or HBsAg conversion among 33 patients, and evidence of ALT elevation only in one patient who had been massively transfused.

### SURGICAL PROCEDURE

Following Whipple resection, the remaining ducts are occluded with fibrin sealant. Approximately three to five ml fibrin sealant are injected into the main pancreatic duct, either through a blunt needle or a thin plastic catheter, beginning as deeply as possible in the remaining glandular tissue. As the needle or catheter is slowly withdrawn, fibrin sealant is continously injected until small fibrin thrombi appear on the resection surface. The pancreatic duct is then ligated and the resection area is covered with an additional layer of sealant. Collagen fleece may be applied if hemorrhage is difficult to control (Figures 1–4).

### PATIENTS

This method was employed in 44 consecutively operated patients (25 male, 19 female) undergoing partial pancreaticoduodenectomy for malignancy. There were 5 patients with carcinoma of the common bile duct, 15 with ampullary carcinoma, and 24 patients with carcinoma of the head of the pancreas. The average age was 67.3 years and 74% of the patients were older than 70 years; there was no patient selection.

### RESULTS

There were no perioperative deaths among the 44 patients. One patient developed clinical evidence of persistent necrotizing pancreatitis, necessitating total pancreatectomy. Two patients developed pancreatic fistulas, probably caused by incomplete occlusion. With conservative therapy, these receded within 2 to 4 weeks post-surgery. Technical short-comings in these three patients, treated early in our series probably led to incomplete occlusion and, as a consequence, retention pancreatitis.

**Figure 1** Duct occlusion with Fibrin Sealant. (*See colour plate at the back of this issue.*)

In all patients, we noted a moderate elevation of serum amylase levels postoperatively (400 + 100 IE/l), which subsided after one week.

All patients lost their exocrine function. To compensate for the loss of pancreatic enzymes, all surviving patients are given pancreatic granules as a dietary supplement.

With regard to pancreatic endocrine function, all the patients with normal glucose tolerance before surgery were unchanged postoperatively. Among those 6 patients with latent diabetes, demonstrated by abnormal preoperative glucose tolerance tests, 3 developed manifest diabetes post surgery. Among these patients

**Figure 2** Ligature of the occluded duct. (*See colour plate at the back of this issue.*)

the manifest diabetes can be also attributed to resection of intact pancreatic tissue.

After an observation period of 2 years, 14 of the surviving patients have normal fasting blood sugar levels; 6 of these patients have normal fasting blood sugar after 3 years.

In a control series of 10 patients, pancreatic duct ligation was performed without fibrin sealant occlusion. In this group, 2 patients died of necrotizing pancreatitis, and 2 patients developed pancreatic fistulae. In ten consecutive patients treated at our hospital by partial pancreaticoduodenectomy with pancreaticojejunal anasto-

**Figure 3** Sealing of the resection surface with Fibrin Sealant. (*See colour plate at the back of this issue.*)

mosis, there were three deaths and four patients had significant perioperative morbidity. Thus, the results obtained with pancreatic duct occlusion using fibrin sealant were clearly superior to those seen either with simple duct ligation, or with pancreaticojejunostomy in our patient population (Table 2).

### DISCUSSION

The variety of techniques employed in performing Whipple's operation indicate the surgeons general dissatisfaction with the results of partial pancreaticoduodenectomy for malignant indications. Even experts in the use of anastomotic techniques encounter significant postoperative insufficiency rates. Of 824 cases reported in

**Figure 4** Sealing in combination with collagen fleece. (*See colour plate at the back of this issue.*)

**Table 2** Complication and mortality rates following pancreaticoduodenectomy – with and without use of fibrin sealant

| | *Number of Patients* | *Perioperative Mobidity* | *Perioperative Mortality* |
|---|---|---|---|
| Fibrin sealant occlusion | 44 | 3 (6, 8%) | 0 |
| Duct ligation | 10 | 2 (20%) | 2 (20%) |
| Pancreaticojejunal anastomosis | 12 | 4 (33%) | 3 (25%) |

Japan, Nakase[4] noted an incidence of dehiscence at the pancreaticojejunostomy site of 14%, while other series report the incidence as being between 11% and 23%[5–8]. Dehiscence of the pancreaticoenteric anastomosis constitutes the most frequent postoperative complication associated with partial pancreaticoduodenectomy. What is more, it accounts for a high percentage of the perioperative deaths.

Our objective was to minimize postoperative pancreatitis and fistula formation following pancreaticoduodenectomy, by using an atraumatic and fluid-tight procedure, while maintaining endocrine function via conservation of the pancreatic tail. By the use of a low-viscosity fibrin sealant we are able to ensure the complete occlusion of the duct system. The results we have described are very similar to those of Ascherl and co-workers who conducted a comparative study which demonstrated the advantages of fibrin sealing[19].

Occlusion of the pancreatic duct with prolamin does not yield uniformly favorable long term results, probably because the high and variable viscosity of this material hampers complete occlusion of the collateral branches[19]. Furthermore, occlusion with prolamin is usually followed by interstital fibrosis with subsequent high-grade sclerosis of the exocrine pancreatic parenchyma beginning on the second postoperative day, with significant alteration of the endocrine cell function[20].

The result is a worthwhile longterm result in endocrine function[21]. The disadvantage of the fibrin-occlusion without anastomosis is the complete loss of exocrine function and the continous need for treatment with pancreatic enzymes.

In the 44 patients in whom we have used fibrin sealant, we were able to reduce significantly the perioperative mortality as well as the postoperative complication rate of partial pancreaticoduodenectomy by employing fibrin sealant occlusion of the remaining pancreatic ducts. We continue to see satisfactory long-term results, due to less interstitial reaction and fibrosis with this physiologic substance which doesn't induce a foreign body reaction.

Recently we have performed abdominal CAT scans of twelve patients. They demonstrated that the pancreas remaining -- 3 years postoperatively -- has a delicate parenchyma, which appears quite normal.

Simple handling and the atraumatic approach justify routine use of this method.

*References*

1. Schapiro, T.M. (1975) Adenocarcinoma of the pancreas: a statistical analysis of biliary bypass vs Whipple resection in good risk patients. *Ann. Surg.*, **182**, 715–721
2. Nakase, A., Matsumoto, Y., Uchida, K. and Honjo, I. (1977) Surgical treatment of cancer of the pancreas and the periampullary region. Cumulative results in 57 institutions in Japan. *Ann. Surg.*, **185**, 52
3. Warren, K.W., Christophi, C., Armendariz, R. and Basu, S. (1983) Current trends in the diagnosis and treatment of carcinoma of the pancreas. *Amer. J. Surg.*, **145**, 813–818
4. Kummerle, F. and Ruckert, K. (1984) Surgical Treatment of Pancreatic Cancer. *World J. Surg.*, **8**, 889–894
5. van Herden, J.A. (1984) Pancreatic Resection for Carcinoma of the Pancreas: Whipple Versus Total Pancreatectomy -- An Institutional Perspective. *World J. Surg.*, **8**, 880–888
6. Grace, P.A., Pitt, H.A., Tompkins, R.K., Denbentsen, L. and Longmire, W.P. Jr. (1986) Decreased morbidity and mortality after pancreatoduodenectomy. *Amer. J. Surg.*, **151**, 141–148
7. Tarazi, R.Y., Hermann, R.E., Vogt, D.P., Hoerr, S.O., Esselstyn, C.B. Jr. *et al.* (1986) Results of surgical treatment of periampullary tumors: a thirty-five-year experience. *Surgery*, **100**, 716–722
8. Christ, D.W., Sitzmann, J.V., Cameron, J.L. (1987) Improved hospital morbidity, mortality, and survival after the Whipple procedure. *Ann. Surg.*, **206**, 358–365
9. Trede, M. and Schwall, G. The complications of pancreatectomy. *Ann. Surg.*, **207**, 39–47
10. Warshaw, A.L. and Swanson, R.S. (1988) Pancreatic cancer in 1988. Possibilities and probabilities. *Ann. Surg.*, **208**, 541–552
11. Funovics, J. and Wenzel, E. (1985) Duodenopankreatektomie: Anastomosierung nicht notwendig. *Lang Arch. Chir.*, **366**, 613
12. Marczell, A.P., Dufek, H. and Hold, H. (1980) Erfahrungen bei der Blutstillung mittels Fibrinklebers in der Abdominalchirurgie. *Wiener Klin. Wschr.*, **82**, 807
13. Marczell, A.P. (1985) Experiences with Fibrin Glue in Pancreas Surgery. *Akt. Chir.*, **20**, 52–54
14. Moossa, A.R., Scott, M.H. and Lavelle-Jones, M. (1984) The Place of Total and Extended Pancreatectomy in Pancreatic Cancer. *World J. Surg.*, **8**, 895–899
15. Pederzoli, P., Bassi, C., Falconi, M., Albrigo, R. Vantini, I. and Micciolo, R. (1986) Conservative treatment of external pancreatic fistulas with parenteral nutrition alone or in combination with intravenous infusion of somatostatin, glucagon or calcitonin. *Surg., Gynecol. Obstet.*, **163**, 428–432

16. Joehl, R.J., Nahrwold, D.L. (1985) Inhibition of human pancreatic secretion by terbutaline as a potential agent for treating patients with pancreatic fistula. *Surg. Gynecol. Obstet.*, **160**, 109–114
17. Land, W., Illner, W-D., Abendroth, D. and Landgraf, R. 91984) Experience with 13 segmental pancreas transplants in cyclosporine-treated diabetic patient using Ethibloc for duct obliteration (surgical aspects). *Transplant Proc.*, **16**, 729–733
18. Rossi, R.L., Soeldner, J.S., Braasch, J.W., Heiss, F.W., Shea, J.A. *et al.* (1986) Segmental pancreatic autotransplantation with pancreatic ductal occlusion after near total or total pancreatic resection for chronic pancreatitis. *Ann. Surg.*, **203**, 626–635
19. Ascherl, R., Hirmer, H. *et al.* (1985) Pankreasgangokklusion mit Fibrin. Kongressberight der 26. *Tagung der östrr Gesellschaft fürr Chir*, S 159
20. Grossner, D., Koge, H.V., Berkoff, M., Klapder, F. and Klöppel, G. (1989) Insulinreserve und Morphologie des Pankreasschwanzes nach Pankreas-kopfresektion bei unterschiedlichen Methoden der Versorgung. *Langenbeck's Arch. Chir.*, **374**, 4–11
21. Lorenz, D., Wolff, H. and Waclawiczek, H.W. (1988) Die Pankreas-gangocclusion in der Resektionsbehandlung der chronischen Pankreatitis und des Pankreaskopfcarcinoms. Eine 3jährige Nachbehandlungsstudie. *Chirurg.*, **59**, 90

### INVITED COMMENTARY

Despite the remarkable decrease in operative morbi-mortality of pancreatoduodenectomy in specialized centers, complications from the pancreatojejunostomy remains a concern. Patients with soft glands and pancreatic ducts of small diameter are at high risk for developing complications. Techniques to attempt to minimize these complications include the use of invaginating dunking techniques, the use of a separate Roux-en-Y for the pancreatic anastomosis, the use of stents, the injection and occlusion of the pancreatic duct with different polymers, the prophylactic use of Somastatin, etc. However, it is likely that surgical expertise with pancreatoduodenectomy as developed in specialized centers is the primary reason for the decrease in the complication rate from the pancreatojejunostomy. Nevertheless, in the best of hands, the soft pancreas with a small pancreatic duct continues to represent a major challenge. The experience with fibrin sealant for occlusion of the pancreatic duct in the use of a nonanastomosed pancreatic stump technique of pancreatoduodenectomy provides another alternative. Using this technique, the authors reported no operative mortality in 44 patients, while the 22 patients that had either duct ligation or pancreatojejunal anastomosis had a mortality of 23%. This latter mortality, though, is very high for current standards and, therefore, the difference with the fibrin sealant occlusion group appears more significant than if compared to the mortality of less than 5% reported by other centers.

Although the data on endocrine function presented here is limited, it would tend to support the idea that ductal occlusion in humans may have less effect on long-term endocrine function than that reported in the animal model. However, for an accurate assessment of the progressive loss of endocrine function as compared to a sutured anastomosis, a prospective randomized trial would be needed.

The technique presented here should be looked upon as a possible option that requires further assessment. The potential benefits of the fibrin sealant combined with a suture anastomosis, either of the mucosa-to-mucosa or of the dunking type would be of interest.

Ricardo L. Rossi
Lahey Clinical Medical Centre
41 Mall Road
Burlington
Mass 01805
USA

